# Stromal cells from perinatal and adult sources modulate the inflammatory immune response in vitro by decreasing Th1 cell proliferation and cytokine secretion

**DOI:** 10.1002/sctm.19-0123

**Published:** 2019-10-22

**Authors:** Oula Khoury, Anthony Atala, Sean V. Murphy

**Affiliations:** ^1^ Wake Forest Institute for Regenerative Medicine Wake Forest School of Medicine Winston‐Salem North Carolina

**Keywords:** cytokines, immunosuppression, lymphocytes, stem cells

## Abstract

Many immune‐mediated conditions are associated with a dysregulated imbalance toward a Th1 response leading to disease onset, severity, and damage. Many of the therapies such as immunomodulators or anti‐TNF‐α antibodies often fall short in preventing disease progression and ameliorating disease conditions. Thus, new therapies that can target inflammatory environments would have a major impact in preventing the progression of inflammatory diseases. We investigated the role of human stromal cells derived from the amniotic fluid (AFSCs), the placenta (PLSCs), and bone marrow‐derived mesenchymal stromal cells (BM‐MSCs) in modulating the inflammatory response of in vitro‐stimulated circulating blood‐derived immune cells. Immune cells were isolated from the blood of healthy individuals and stimulated in vitro with antigens to activate inflammatory responses to stimuli. AFSC, BM‐MSCs, and PLSCs were cocultured with stimulated leukocytes, neutrophils, or lymphocytes. Inflammatory cytokine production, neutrophil migration, enzymatic degranulation, T cell proliferation, and subsets were evaluated. Coculture of all three stromal cell types decreased the gene expression of inflammatory cytokines and enzymes such as IL‐1β, IFN‐γ, TNF‐α, neutrophil elastase, and the transcription factor NF‐κB in lipopolysaccharide‐stimulated leukocytes. With isolated phytohemagglutinin‐stimulated peripheral blood mononuclear cells, cells coculture leads to a decrease in lymphocyte proliferation. This effect correlated with decreased numbers of Th1 lymphocytes and decreased secreted levels of IFN‐γ.

## Graphical abstract

Perinatal cells suppress the proliferation of Th1 cells and their associated cytokines.

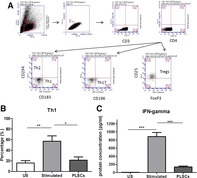

**Significance statement**

This study highlights the immunosuppressive properties of perinatal cells on Th1 cells and their associated cytokines thus providing further understanding of the role of perinatal cells as a potential therapy to target Th1 mediated diseases.

## 1 INTRODUCTION

Inflammation is a tightly regulated phenomenon that requires coordination of cytokine signaling.^1^ Cytokines involved in the inflammatory response are most abundantly produced by T helper cells (Th), a subtype of T lymphocytes that are distinguished by expressing the surface marker CD4, as opposed to effector T lymphocytes that express the surface marker CD8. Th lymphocytes are further subdivided into several subsets, including Th1 and Th2, each with distinct roles in inflammation. Th1 cells have pro‐inflammatory properties, mainly secrete IFN‐γ, IL‐2, and TNF‐α and aid in the fight against intracellular bacterial and parasitic infections. Th2 cells, on the other hand, produce the cytokines IL‐4 and IL‐5, mediate an eosinophilic response that activates the production of immunoglobulin (Ig)‐E, and have been implicated in allergic reactions. Th2 also produce IL‐10, thus, potentially having some anti‐inflammatory properties. A balance between Th1 and Th2 responses and their associated cytokines are thus important to maintain a balanced immune response.^2^, ^3^, ^4^, ^5^ Dysfunction in the regulation of Th1/Th2‐mediated inflammation can lead to disease and tissue damage.^6^ For example, in chronic obstructive pulmonary disease, lung tissue‐derived elastin is responsible for the activation of CD4^+^ Th1 cell‐mediated inflammation, causing tissue injury and emphysema.^7^ Crohn's disease (CD) is also closely related to a Th1 skewed immune response, in which enhanced IFN‐γ expression and release have been shown.^8^, ^9^

Th1‐mediated inflammation is also implicated for many types of autoimmune diseases, including rheumatoid arthritis (RA), multiple sclerosis (MS), corneal transplant rejection, and type I diabetes.^10^, ^11^, ^12^, ^13^ Current treatments for these types of diseases include the use of Th1 pre‐inflammatory cytokine antagonists, such as antibodies to TNF‐α or IFN‐γ.^14^ However, these treatments, even when used in combination, appear to have limited effects on the more chronic and severe forms of disease and there is a pressing need for the development of new treatment strategies. In this study, we investigated the potential role of stromal cells derived from different tissue sources to modulate the inflammatory response of immune cells in vitro, thus identifying a potential anti‐inflammatory therapy for a range of diseases.

Stromal cells present in cell populations derived from perinatal tissues such as the placenta and amniotic fluid, offer an advantage over other sources like bone marrow (BM) because of their ease of collection, ready availability, high abundance, and high proliferation rates.^15^, ^16^ Human amnion‐derived mesenchymal stromal cells (hAMSC) have been shown to skew macrophage polarization toward M2, inhibit monocyte differentiation into dendritic cells, and reduce the expression of Th1, Th2, and Th17 associated markers and their corresponding subset cytokines including TNF‐α, IFN‐γ, IL‐1β, IL‐5, IL‐9, and IL‐22.^17^ In another study, human umbilical cord‐derived MSCs (UC‐MSCs) inhibited the proliferation of lymphocytes stimulated with phytohemagglutinin (PHA) under coculture setting in vitro, and enhanced the abundance of T regulatory (Tregs) cells, while also increasing IL‐10 levels.^18^ Thus, perinatal cells exhibit a wide range of therapeutic properties that make them the ideal candidates to treat inflammatory diseases. In this study, we compared the potential immunomodulatory properties of amniotic fluid‐derived cells (AFSCs), placenta‐derived cells (PLSCs), and BM‐derived mesenchymal stem cells (BM‐MSCs) on antigen‐stimulated leukocytes, lymphocytes, neutrophils, and T cell subsets derived from the blood of healthy donors.

## 2 MATERIALS AND METHODS

### 2.1 Patient selection and sampling

This study and all procedures described in this proposal were approved by the Wake Forest Baptist Medical Center ‐ Institutional Review Board (IRB) (Study ID: IRB000207670). Healthy donors were identified, consented, and recruited by our study coordinators. They had 15 mL blood drawn by a clinical phlebotomist at the Wake Forest Baptist Medical Center. Donors were adults (above 18 years old). At the time of recruitment, donors did not present with a history of blood or bleeding disorders. Data collected on each subject include age and gender. The in vitro immunologic assays were performed at Wake Forest Institute of Regenerative Medicine. Except for the time during which blood is withdrawn, the subjects were not involved in any research procedure.

### 2.2 Isolation and culture of stromal cells

Stromal cells derived from human amniotic fluid and placental tissue were isolated, characterized, and cryopreserved in the Regenerative Medicine Clinical Center located within the Wake Forest Institute for Regenerative Medicine (WFIRM). These cell lines have been developed under current Good Manufacturing Practices (cGMP) in accordance with regulations for clinical applications and were banked and characterized under FDA guidelines for rapid translation of research into clinical trials. BM‐MSCs were isolated using the anti‐Stro −1 antibody (R&D Systems, Minneapolis, Minnesota) and magnetic sorting (Miltenyi Biotec, Auburn, California) as previously described.^19^, ^20^, ^21^ Cells were then grown until 70% confluence in gelatin (Sigma‐Aldrich, St. Louis, Missouri) coated flasks and MSCGM (Lonza, Basel, Switzerland) supplied with 100 U/mL penicillin/streptomycin (Gibco‐Life Technologies, Carlsbad, California). PLSCs were manufactured by WFIRM and in the Regenerative Medicine Clinical Center. PLSCs were isolated from the chorionic tissue of the placenta via enzyme digestion. Isolated placenta‐derived cells were subsequently plated in Alpha‐MEM (Lonza) supplemented with AmnioMax (Thermo Fisher Scientific, Waltham, Massachusetts) media and Glutamax (Gibco‐Life Technologies) to obtain PLSCs. Cells were passaged with TrypLE when the cultures were 70% confluent until sufficient cells were obtained. c‐kit positive cells were selected by Miltenyi device and maintained in culture until sufficiently expanded. Cells were cryopreserved for use in experiments at passage 5 as a Master Cell Bank. Prior to cryopreservation, cells were tested for sterility, endotoxin, and mycoplasma which were negative. Karyotype was tested and found to be normal. AFSCs were isolated and cultured as previously described.^22^ Both perinatal cells are c‐kit^+^, have a doubling time of approximately 36 hours and are nontumorigenic. AFSCs and PLSCs retain long telomeres and express embryogenic stem cell markers (ie, Sox2 and OCT4). They are highly multipotent and can differentiate into cells of all three germ layers. AFSCs and PLSCs, like BM‐MSCs, also expressed adult cell surface markers including CD44, CD90, CD105, and CD166. AFSCs and PLSCs were cultured in alpha MEM supplied with Chang media (Fujifilm Irvine Scientific, Santa Ana, California), and AmnioMax media respectively, and were supplied with 18% fetal bovine serum and 1% penicillin/streptomycin. All cells were grown at 37°C and 5% CO_2_ in a humidified atmosphere. Cells between passages 10‐15 were used. Prior to use, cryopreserved cells were thawed and cultured for 4 days in their respective media. TrypLE was used for the initial isolation and expansion of the cell stocks, whereas 0.05% trypsin was used for routine cell culture. So, cells were detached using 0.05% or 0.25% (for BM‐MSCs) trypsin and viability was assessed using trypan blue.

### 2.3 Leukocyte isolation from peripheral blood

Total white cells (leukocytes) were isolated by HetaSep (StemCell Technologies, Vancouver, Canada) density gradient centrifugation. Briefly, 1‐part HetaSep was added to five parts blood and the sample was centrifuged at 90*g* for 3 minutes at room temperature. The supernatant rich in total white cells was then collected and washed three times with Roswell Park Memorial Institute (RPMI) 1640 media +1% bovine serum albumin (BSA). Red blood cell (RBC) lysis was performed by incubating the leukocyte pellet in 10 mL of RBC Lysing buffer Hybri‐Max (Sigma‐Aldrich) at room temperature for 10 minutes with frequent vortexing. Cell count and viability was determined using the trypan blue exclusion method.

### 2.4 Isolation of peripheral blood mononuclear cells (PBMCs)

PBMCs were isolated from blood by density centrifugation using Histopaque (Sigma). Briefly, the total volume of blood was laid over an equivalent volume of Histopaque and the sample was centrifuged at 450*g* for 30 minutes. PBMCs were then collected from the buffy coat phase and transferred into a fresh tube and washed with PBS and centrifuged at 770*g* for 10 minutes. RBC lysis was performed by incubating the PBMC pellet in 10 mL of RBC Lysing buffer Hybri‐Max (Sigma‐Aldrich) at room temperature for 10 minutes with frequent vortexing. Cell count and viability was determined using the trypan blue exclusion method.

### 2.5 Neutrophil isolation

Neutrophils were isolated by means of negative selection magnetic isolation using the Human Neutrophil Enrichment Kit (StemCell Technologies) according to the manufacturer's guidelines. Briefly, total white cells were incubated with EasySep Human Neutrophil Enrichment cocktail for 10 minutes at 4°C. EasySep nanoparticles were then added to the mixture and were incubated for 10 minutes at 4°C. The suspension was then mixed and brought to a total volume of 2.5 mL. The tube was then inserted into the EasySep magnet, and with one continuous motion, the tube was inverted, and the contents were poured into a fresh 5 mL polystyrene tube. Cell count and viability were determined using the trypan blue exclusion method.

### 2.6 Lymphocyte proliferation assay

PBMCs were stimulated with 10 μg/mL PHA and seeded at 20 × 10^4^ cells in a 96‐well plate in the presence or absence of 20 × 10^3^ mitomycin‐C (25 μg/mL) treated AFSCs, BM‐MSCs, or PLSCs. Stromal cells were plated into a 96‐well plate and allowed to adhere overnight. The coculture system was incubated for 6 days before cell proliferation was assessed using the Cell proliferation ELISA bromodeoxyuridine (BrDU) kit (Roche, Basel, Switzerland). An equivalent of 0.1 mM BrDU was added on day 5 for an overnight incubation.

### 2.7 Flow cytometry T cell subset analysis

PBMCs were collected and stained with cell surface markers CD3 (0.0125 μg/μL), CD4 (0.00625 μg/μL) CD294 (0.05 μg/μL) (Th2), CD183 (0.05 μg/μL) (Th1), CD196 (0.1 μg/μL) (Th17), and CD25 (0.0125 μg/μL) (Tregs) obtained from BD Biosciences (San Jose, California). PBMCs were incubated for 30 minutes, then washed with PBS twice and stored on ice in 1% paraformaldehyde solution until analyzed using a BD Accuri C6 flow cytometer (BD Biosciences). FoxP3 (0.025 μg/μL) intracellular staining was also performed as a marker for the Tregs subpopulation. PBMCs were permeabilized with 0.1% Tween 20 solution for 10 minutes before staining with CD25 and FoxP3 antibodies.

### 2.8 IFN‐γ cytokine ELISA

IFN‐γ secreted levels were measured in the supernatant of cells cultured under the system described above. Briefly PBMCs were centrifuged at 433*g* for 5 minutes, resuspended in PBS for flow analysis whereas the supernatants were collected and assessed for IFN‐γ protein levels using the Human IFN Gamma PicoKine ELISA Kit (Boster, Pleasanton, California) following the manufacturer's protocol.

### 2.9 Th1/Th2/Th17 cytokine bead assay

Supernatants collected from cells cultured under the system described above were used to measure the levels of seven cytokines by flow cytometry, IL‐2, IL‐4, IL‐6, IL‐10, TNF, IFN‐γ, and IL‐17A using the Human Th1/Th2/Th17 Kit (BD Biosciences), according to the manufacturer's protocol. Analysis was acquired using a BD Accuri C6 flow cytometer (BD Biosciences).

### 2.10 Neutrophil migration assay

Isolated neutrophils were seeded at a density of 25 × 10^4^ cells in the top chambers of a 96‐well plate with a transwell system in the presence (coculture) or absence of 2 × 10^4^ stromal cells. Cells were incubated for 4 hours before neutrophil migration was assessed in response to 80 ng/mL IL‐8 solution in the bottom chamber of the transwell system, using the Cytoselect 96‐well cell migration assay 3 μm, fluorometric format (Cell Biolabs, Inc, San Diego, California), according to the manufacturer's protocol. Briefly, neutrophils were allowed to migrate from the upper chamber through the polycarbonate membrane, where they attach to the bottom side. The migratory cells were dissociated from the membrane by the addition of Cell Detachment Buffer to the lower chamber. Neutrophils were lysed and quantified using the CyQuant GR Fluorescent dye and fluorescence was measured using a fluorescent microplate reader at Ex/Em = 480 nm/520 nm.

### 2.11 Myeloperoxidase (MPO) and neutrophil elastase (NE) activity assays

Isolated neutrophils were stimulated with 100 nM phorbol myristate acetate and seeded at a density of 25 × 10^4^ cells in a 96‐well plate in the presence or absence of 20 × 10^3^ stromal cells. Cells were cultured for 2 hours and 30 minutes before MPO and NE activities were assessed using the Neutrophil Myeloperoxidase Activity Assay kit (Cayman Chemical, Ann Arbor, Michigan) and Neutrophil Elastase Activity Assay Kit (Abcam, Cambridge, Massachusetts). MPO activity was assessed by incubating the cells supernatant with MPO substrate for 10 minutes. Absorbance was read using a plate reader at 650 nm. NE activity was assessed by incubating with NE substrate for 20 minutes and measuring the output on a fluorescent microplate reader at Ex/Em = 380/500 nm.

### 2.12 In vitro stimulation of total white cells

Total white cells (2 × 10^6^ cells) were stimulated with 10 ng/mL lipopolysaccharide (LPS) for 24 hours in a 12‐well plate, supplied with RPMI +1% BSA media. LPS stimulated cells were exposed to 20 × 10^4^ stromal cells in a transwell culture system using cell culture inserts (Millicell cell culture inserts 0.4 μm, 12 mm diameter). Cells were used to perform the assays detailed below.

### 2.13 Cytokine ELISA

Cell supernatants from the in vitro stimulated and unstimulated total white cells were collected by centrifuging the total white cells at 433*g* for 5 minutes. NE, IL‐6, and TNF‐α protein levels were then assessed using the Human PMN Elastase Platinum ELISA (Invitrogen, Carlsbad, California), IL‐6 and TNF‐α PicoKine ELISA Kits (Boster).

### 2.14 RNA sampling, cDNA generation, and qRT‐PCR

RNA was isolated using the TRIzol reagent (Invitrogen) method according to the manufacturer's guidelines. RNA quality was determined using the NanoDrop 2000 Spectrophotometer (Thermo Fisher Scientific). cDNA was generated using the High Capacity cDNA Reverse Transcriptase Kit (Applied Biosystems, Foster City, California) according to the manufacturer's protocol. qRT‐PCR was performed using Taqman Primers for IL‐8, IL‐1β, TNF‐α, NE, IL‐6, NF‐κB, IFN‐γ, CCL2, and IL‐10 (Thermo Fisher Scientific) following the TaqMan Fast Advanced Master Mix Protocol (Applied Biosystems).

### 2.15 Nuclear extraction and NF‐kB ELISA

Cells from the above cell culture system were collected and centrifuged at 433*g* for 5 minutes. The cell pellet was used for nuclear and cytoplasmic extraction using the NE‐PER nuclear and cytoplasmic extraction reagents kit (Abcam) following the manufacturer's protocol. Briefly, cells were incubated with cytoplasmic extraction reagent (CER) and vortexed vigorously for 5 seconds. Tubes were then centrifuged at maximum speed (16 000*g*) for 5 minutes to obtain the supernatant containing the cytoplasmic extract. The remaining pellets were incubated with the nuclear extraction reagent (NER) included in the kit and vortexed vigorously for 15 seconds. After centrifugation at maximum speed (16 000*g*) for 10 minutes, nuclear extracts in the supernatant were collected and stored at −80°C until use. Protein quantification was performed on nuclear extracts using the Pierce BCA Protein Assay Kit (Thermo Fisher). Nuclear extracts were then used to perform the NF‐kB p50 transcription factor assay kit (Abcam) following the kit's protocol.

### 2.16 Statistical analysis

In vitro assays were performed with triplicate technical replicates, and data from the groups were pooled and displayed as mean ± SEM. Data were analyzed using GraphPad Prism version 6.0. One‐way ANOVA tests were used to determine statistical significance between the groups of immune cells at baseline (unstimulated), exposed to stimulus and/or perinatal cells. Tuckey's test was performed to correct for multiple comparisons. A confidence limit of 95% was considered significant. In Figures, bars represent SEM. *P < .05; **P < .01; ***P < .001.

## 3 RESULTS

### 3.1 Assessment of stromal cells' effect on gene expression profile of inflammatory cytokines and enzymes

**Figure 1:**
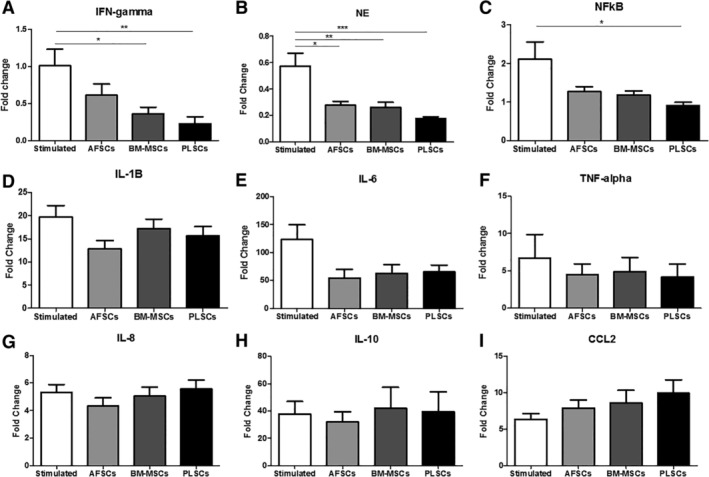
Gene expression of leukocytes at baseline and following stimulation with 10 ng/mL lipopolysaccharide (LPS), in the presence and/or absence of stromal cells. A, Interferon gamma (IFN‐γ); B, neutrophil elastase (NE); C, nuclear factor kappa B (NF‐κB); D, interleukin 1β (IL‐1β); E, interleukin‐6 (IL‐6); F, tumor necrosis factor‐alpha (TNF‐α); G, interleukin‐8 (IL‐8); H, interleukin‐10 (IL‐10); and I, chemokine ligand‐2 (CCL2). Bars represent SEM. *P < .05; **P < .01; ***P < .001

A total of 81 samples (obtained from 21 females and 60 males, average age: 33, age range: 23‐57) were used in this study. We first compared the gene expression profile of healthy leukocytes following LPS stimulation with that of the LPS‐stimulated leukocytes cultured with the three stromal cell types located in the upper chamber of a transwell system for indirect coculture conditions. RNA isolation was performed 24 hours after initiation of LPS stimulation for all groups. We found that BM‐MSCs (*P* < .05) and PLSCs (*P* < .01) coculture decreased IFN‐γ gene expression in LPS‐stimulated leukocytes (Figure 1A). A significant decrease in the NE (Figure 1B) gene expression was also observed with the transwell coculture of AFSCs (*P* < .05), BM‐MSCs (*P* < .01) as well as PLSCs (*P* < .001). We also observed a significant decrease in NF‐κB gene expression only in the presence of PLSCs (*P* < .05) (Figure 1C). AFSCs and BM‐MSCs coculture also showed a trend of decreased NF‐κB gene expression; however, this was not statistically significant. We observed decreased levels of IL‐6 and TNF‐α with the transwell coculture of all stromal cell types, however these changes were not statistically significant. We did not observe any changes in IL‐8, IL‐10, or CCL2 expression levels with any of our stromal cell types (Figure 1G‐I). Although some differences between the three stromal cell populations were observed, these findings indicate that these stromal cells have an inhibitory effect on inflammatory cytokine gene expression, including the NF‐κB signaling pathway, and downstream factors such as NE. These data also indicate that this phenomenon is not dependent on direct contact between stromal cells and leukocytes, suggesting a role of soluble factors produced by stromal cells in transwell coculture.

### 3.2 Effect of stromal cells on cytokine release profiles

**Figure 2:**
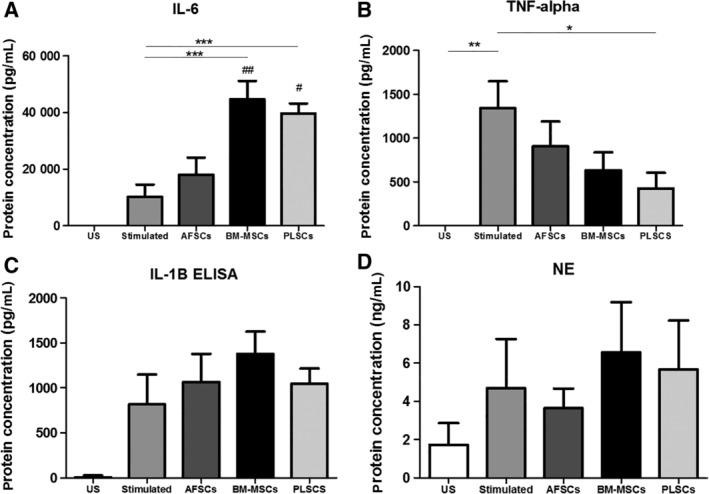
Secreted levels of cytokines and enzymes from unstimulated (US) leukocytes and following stimulation with lipopolysaccharide (LPS) alone, and in the presence of amniotic fluid‐derived cells (AFSCs), bone marrow‐derived mesenchymal stromal cells (BM‐MSCs), and placenta‐derived cells (PLSCs). A, Interleukin‐6 (IL‐6); B, tumor necrosis factor‐alpha (TNF‐α); C, interleukin‐1 beta (IL‐1 β); and D, neutrophils elastase (NE). Bars represent SEM *P < .05; **P < .01; ***P < .001; #*P* < .05 compared to AFSCs, ##*P* < .01 compared to AFSCs

Next, we assessed cytokine protein levels in the cell culture supernatants. Cytokine ELISAs were performed on supernatants obtained from healthy leukocytes following LPS stimulation and compared to LPS‐stimulated leukocytes cocultured with stromal cells located in the upper chamber of a transwell system. Culture supernatants were collected (combined from the lower and upper chambers) 24 hours after initiation of LPS stimulation for all groups. Protein levels of NF‐κB in cell extracts and protein levels of NE, IL‐8, IL‐10, CCL2, and IFN‐γ in cells supernatants did not show any significant differences with LPS stimulation, or in any of coculture groups (data not shown). Levels of IL‐6 in the supernatant derived from leukocytes increased following LPS stimulation, and coculture with all three stromal cell populations further increased the levels of secreted IL‐6, however only BM‐MSCs and PLSCs coculture were statistically significant (*P* < .001) (Figure 2A). PLSCs coculture significantly decreased the protein levels of secreted TNF‐α following LPS stimulation (*P* < .05). IL‐1β levels in the supernatant seem to increase with the transwell coculture of all stromal cell populations; however, changes were not statistically significant (Figure 2C). Secreted NE levels were unaffected by the LPS stimulation and remained unchanged with coculture of all stromal cell populations. In comparison to gene expression analysis, in which RNA was isolated only from leukocytes in the lower transwell chamber, analysis of the cytokines in the culture supernatant, can represent cytokines secreted by leukocytes and potentially also by the cocultured stromal cell populations. It appears the primary effect of LPS‐stimulation was the secretion of inflammatory cytokines IL‐6, TNF‐α, IL‐1β, and IL‐10, which are all known to be secreted by LPS‐stimulated monocytes. Although stromal cell coculture appeared to decrease secreted TNF‐α levels, the observed increase in IL‐6 may be due to increased secretion from monocytes, and/or secretion from the stromal cells themselves, which have been shown to secrete IL‐6 under various conditions.^23^

### 3.3 Effect of stromal cells on neutrophil migration and enzymatic activities

**Figure 3:**
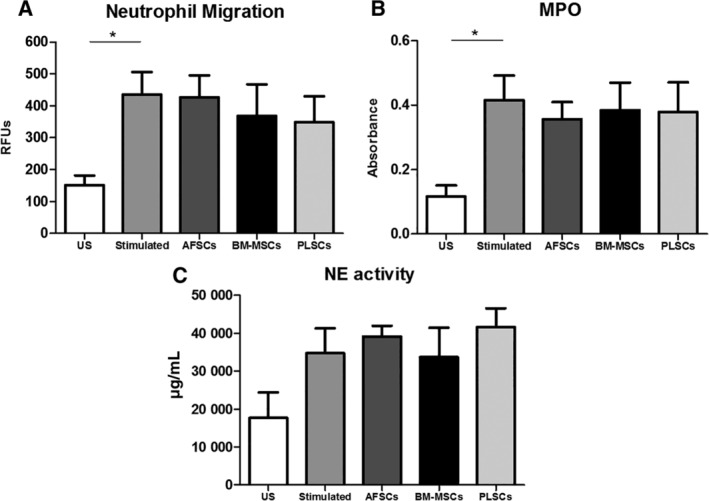
Effect of amniotic fluid‐derived cells (AFSCs), bone marrow‐derived mesenchymal stromal cells (BM‐MSCs), and placenta‐derived cells (PLSCs) on isolated neutrophils' migration and enzymatic properties. A, Neutrophil migration to an IL‐8 gradient; B, myeloperoxidase (MPO) activity; and C, neutrophils elastase (NE) enzymatic activity assays. Bars represent SEM. *P < .05

Our qPCR data indicated a significant decrease of NE gene expression following transwell coculture with all three stromal cell types. To explore potential mechanisms of interaction of stromal cells with neutrophils, we isolated neutrophils by means of negative‐selection magnetic isolation and subjected them to an IL‐8 concentration gradient. Neutrophils exhibited a significant increase in migration following exposure to the IL‐8 gradient however; none of the three stromal cell populations had any significant effect on this phenomenon (Figure 3A). Similarly, stromal cells did not seem to affect the degranulation of MPO or NE (Figure 3B,C). These data suggest that there are no significant direct effects of stromal cells coculture on clinically relevant functions of neutrophils under these in vitro conditions.

### 3.4 Lymphocyte proliferation and subset analysis

**Figure 4:**
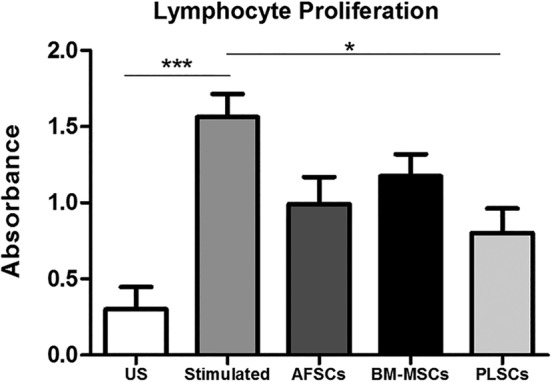
Lymphocyte proliferation assay of phytohemagglutinin (PHA) stimulated healthy derived peripheral blood mononuclear cells (PBMCs) alone and in coculture with amniotic fluid‐derived cells (AFSCs), bone marrow‐derived mesenchymal stromal cells (BM‐MSCs), and PLSCs. Bars represent SEM. *P < .05; ***P < .001

Given that our data showed that coculture with stromal cells had modulatory effects on the gene expression related to T lymphocytes such as NF‐κB, IL‐1β, and TNF‐α, we investigated the effect of all three stromal cell types on lymphocyte proliferation and T cell subsets following antigen stimulation. Peripheral blood‐derived mononuclear cells (PBMCs) were by isolated by Histopaque density centrifugation and stimulated with PHA. Stimulated PBMCs showed significant cell proliferation following 6 days of culture. Direct coculture (both PBMCs and stromal cells in direct contact) of all three cell types decreased the proliferation of stimulated lymphocytes, with a statistically significant effect observed for PLSCs (*P* < .01) (Figure 4).

**Figure 5:**
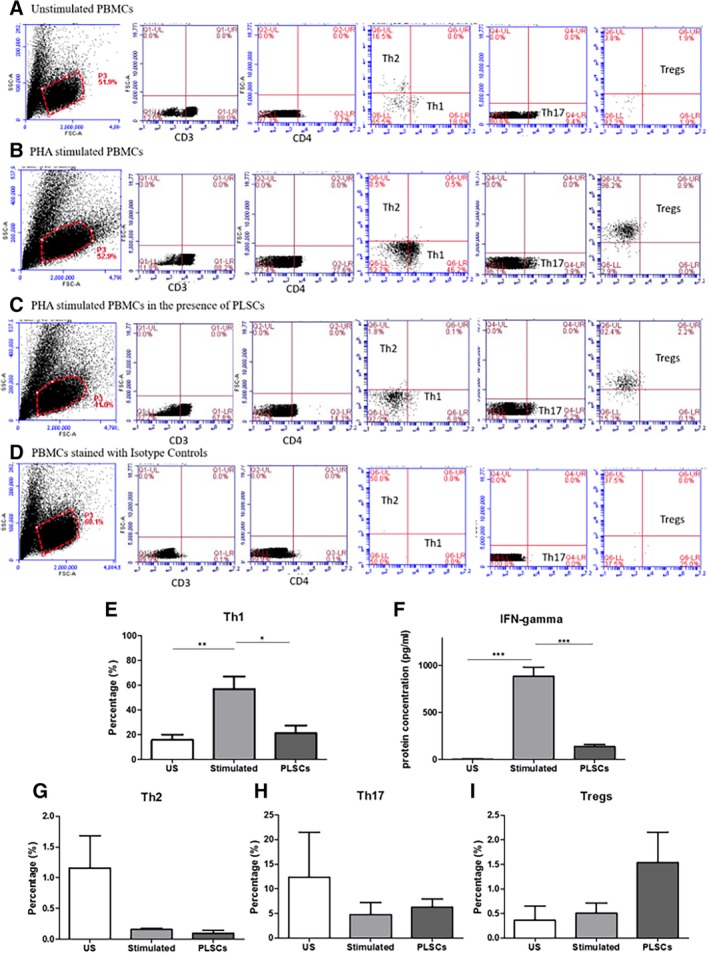
Flow cytometry analysis of CD3+ CD4+ subsets at baseline (unstimulated), following stimulation with phytohemagglutinin (PHA) in the presence and/or absence of placenta‐derived cells (PLSCs). A, Panel showing gating strategy used for analysis of unstimulated peripheral blood mononuclear cells (PBMCs); B, panel showing the gating strategy used for analysis of PHA stimulated PBMCs; C, panel showing the gating strategy used for analysis of PHA stimulated PBMCs in the presence of PLSCs; D, panel showing the gating strategy used for analysis of isotype controls; E, Th1 percentage (%); F, IFN‐γ secreted levels measured in the culture medium; G, Th2 percentage (%); H, Th17 percentage (%); and I, Tregs percentage (%). Bars represent SEM. *P < .05; **P < .01; ***P < .001

Next, we assessed the proportion of each of the CD3^+^ T cell subsets by flow cytometry, distinguishing their phenotype on the basis of cell surface markers and transcription factors: Th1 (CD3^+^ CD4^+^ CD183^+^), Th2 (CD3^+^ CD4^+^ CD294^+^), Th17 (CD3^+^ CD4^+^ CD196^+^), and Tregs (CD3^+^ CD4^+^ CD25^+^ FoxP3^+^). For the subsequent experiments, only PLSCs were used because they exhibited the most widespread reduction of cytokine levels and significant suppression of lymphocyte proliferation. Flow cytometry gating was first performed on the side and forward scatter signals (SSC‐A and FSC‐A) to determine the population of interest and cells were further gated on CD3^+^ CD4^+^ population. This gating strategy was adopted for all cell populations: unstimulated PBMCs and PHA stimulated PBMCs in the presence and/or absence of PLSCs (Figure 5A‐C). We observed that the proportion of Th1 cells significantly increased following stimulation with PHA for 6 days (stimulated) when compared to unstimulated levels (US) (*P* < .01). Coculture with PLSCs significantly decreased the proportion of Th1 cells similar to levels observed in unstimulated PBMCs (*P* < .05) (Figure 5E). We next analyzed the secreted cytokine profile of PMBCs under the same culture conditions. Secreted levels of IFN‐γ in the culture medium coincided with observed Th1 subset proportions, with an observed increase in IFN‐γ levels following stimulation with PHA compared to unstimulated levels (*P* < .001) and a significant reduction of IFN‐γ levels in the presence of PLSCs, when compared to the stimulated group (*P* < .001) (Figure 5F). The presence of PLSCs did not have any significant effect on the percentage of Th2, Th17, or Tregs populations (Figure 5G‐I).

**Figure 6:**
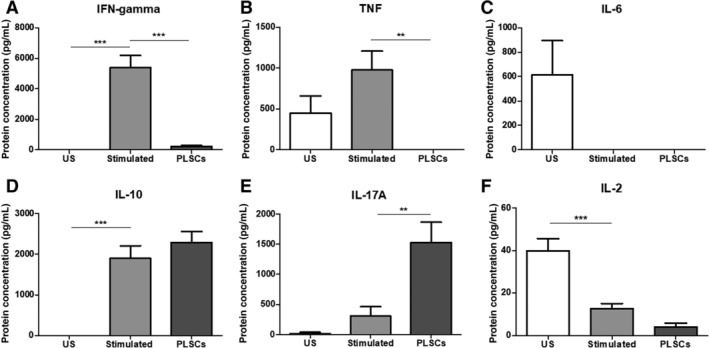
Effects of placenta‐derived cells (PLSCs) on Th1/Th2/Th17 cytokine levels detected in the supernatant of cells at baseline, and after phytohemagglutinin (PHA) stimulation, with and without the presence of PLSCs. A, Interferon‐gamma (IFN‐γ); B, tumor necrosis factor (TNF); C, interleukin‐6 (IL‐6); D, interleukin‐10 (IL‐10); E, interleukin‐17A (IL‐17A); and F, interleukin‐2 (IL‐2). Bars represent SEM. *P < .05; **P < .01; ***P < .001

We also measured the levels of other Th‐associated cytokines using the Th1/Th2/Th17 detection beads. Our findings further confirmed the decreased levels of IFN‐γ in the presence of PLSCs, validating the suppressive effect on Th1 cells. TNF levels were completely abolished in the presence of PLSCs (*P* < .01) when compared to unstimulated and PHA stimulated groups, whereas IL‐6 levels were only detected in unstimulated cells (Figure 6B,C). Both IL‐10 and IL‐17A were elevated following PHA stimulation but PLSCs only increased the levels of IL‐17A compared to the stimulated group (*P* < .01). Levels of IL‐2 measured via ELISA also showed decreased levels after PHA stimulation (*P* < .001) compared to unstimulated cells, and a further reduction was accomplished in the presence of PLSCs when compared to the stimulated cells; however, changes were not statistically significant.

Although we observed varying effects of BM‐MSCs, AFSCs, and PLSCs on the different immune cells and their respective functions, compared to each other, the effects of BM‐MSCs, AFSCs, and PLSCs did not show statistically significant difference. Our data showed potential trends in the effect of the three stromal cell populations on gene expression where it seemed that PLSCs had a more widespread effect on IFN‐γ, NE, and NF‐kB, but changes were not statistically significant. However, the presence of BM‐MSCs (*P* < .01) and PLSCs (*P* < .05) seemed to significantly increase the levels of IL‐6 when compared to AFSCs (Figure 2A). This phenomenon was further supported by the data shown in Supplemental Figure S1 in which we showed that BM‐MSCs and PLSCs secrete significantly higher levels of IL‐6 compared to AFSCs, which did not secrete any detectable levels of IL‐6. This suggests that a key difference between the three cell types is their secreted factors which may have a role in their effects on the different components of the immune system.

## 4 DISCUSSION

This study demonstrated that stromal cells isolated from the amniotic fluid and the placenta have immunomodulatory properties similar to that of BM‐MSCs and thus, may provide a new source for immunomodulatory cell therapy. We evaluated the inhibitory effects of these three cell populations on the cytokine gene expression levels as well as secreted protein levels of LPS‐stimulated leukocytes from healthy donors. LPS is a component that is abundant in Gram‐negative bacteria which triggers an immune inflammatory response by binding to the toll like receptor 4 (TLR4) found on the surface of some immune cells including neutrophils, macrophages and T cells.^24^, ^25^ This induces the activation of transcription factor NF‐κB which subsequently activates the downstream transcription of a plethora of inflammatory cytokines such as IL‐1β, IL‐6, IL‐8, IFN‐γ, and N. We further analyzed the effect of these stromal cells on neutrophils and lymphocytes in isolation, to investigate the probable mechanisms of interaction between the immune cells and perinatal cells. The gene expression data suggested that secreted factors from the stromal cell populations might play a role in modulating cytokine levels, given that the assay was set up under transwell culture conditions. Our findings were in accordance with other studies in showing that perinatal cells, especially PLSCs, decreased the gene expression and protein levels of IFN‐γ and TNF‐α, suggesting that PLSCs may have an inhibitory effect on lymphocytes, particularly Th1 cells.^23^ Furthermore, PLSCs significantly decreased the gene expression of NF‐κB and NE suggesting that PLSCs as well as AFSCs might interact with neutrophils. Moreover, perinatal cells and BM‐MSCs did not have any effect on the migratory or enzymatic activities of isolated neutrophils, despite their inhibitory effect on NE gene expression when total leukocytes were analyzed. This implies that a potential crosstalk between perinatal cells and other components of the immune system is needed to influence these neutrophil functions. PLSCs also decreased the proliferation of Th1 T cell subsets and their associated cytokines.

In our study, we found that PLSCs showed the greatest level of suppression of NF‐κB and NE gene expression. As shown in Figure 1, we also observed a decrease in the gene expression IFN‐γ in the presence of PLSCs. Additionally, PLSCs seem to increase the gene expression of CCL2, a chemokine involved in the recruitment of monocytes. Secreted supernatant cytokine analysis demonstrated increased levels of IL‐6 secretion in the presence of BM‐MSCs and PLSCs, contradicting the gene expression of IL‐6, which remained relatively unchanged. This may be explained by the fact that gene expression analysis was performed only on leukocyte‐derived RNA, with the stromal cells excluded from analysis by their location in the upper transwell membrane. For protein cytokine analysis shown in Figure 2, the analyzed culture supernatant contained proteins that were potentially secreted by both cell types. Stromal cells from the BM, as well as AFSCs and PLSCs have been shown to secrete IL‐6^23^, ^26^ suggesting that the stromal cells might have been the source of the increased IL‐6 levels in our supernatant samples under the transwell culture conditions. This was further supported by our data shown in Supplemental Figure S1, where we evaluated IL‐6 secreted by unstimulated and LPS stimulated AFSCs, BM‐MSCs, and PLSCs. These data demonstrate that both BM‐MSCs and PLSCs secrete IL‐6 into the culture medium and thus represent the likely source of the elevated IL‐6 levels observed in our experimental setting (Supplemental Figure S1). We also observed significantly lower levels of secreted TNF‐α when leukocytes were cocultured with PLSCs. During the early stages of the immune response, IL‐1β, TNF‐α, and IL‐6 are secreted by macrophages and monocytes into the inflammatory milieu, and trigger downstream inflammatory activity including increased vascular permeability and recruitment of neutrophils and lymphocytes.^27^, ^28^, ^29^ Macrophages also promote Th1 mediated immunity by secreting the cytokine IL‐12. IL‐12, along with TNF‐α and other inflammatory cytokines, then, stimulate the production of IFN‐γ.^30^, ^31^ These findings suggest that PLSCs might interact with monocytes and macrophages at early time points and suppress their function to secrete pro‐inflammatory cytokines. This also suggests that the downregulation of these cytokines by PLSCs may also explain the downstream potential inhibitory role of perinatal cells on Th1 cells.^23^, ^31^ Cell supernatants analyzed for NE levels 24 hours after LPS stimulation showed unchanged levels. One limitation of these experiments is that our analysis was performed at a single time‐point, chosen to facilitate detection of a majority of clinically relevant cytokines and acute‐phase reactants. However, this compromise provides only a brief snapshot of the dynamic inflammatory process and is likely to have resulted in our chosen assays missing very early (<60 minutes) as well as late (>48 hours) responses. This may explain some of our findings such as our gene expression data, where the presence of stromal cells had a significant inhibitory effect on the gene expression of NE. NE protein is secreted very early in the inflammatory response, analyzing NE levels after 24 hours might be too late to detect any significant changes, although the effects of NE gene expression under the regulation of the NF‐κB could still be detected. To address these questions, further longitudinal studies are required to provide more information about how perinatal cells interact with both early as well as late‐acting immune responses.

To evaluate potential direct interactions between stromal cells and neutrophils, we purified neutrophils from the total white cells using magnetic bead separation and performed functional assays with stromal cells coculture. In Figure 3, we observed that neutrophil migration toward an IL‐8 gradient, and enzymatic activities remained unaffected by the coculture with stromal cells. Although all three stromal cell population types suppressed the gene expression of NE when evaluated in total leukocytes, this effect was not observed when isolated neutrophils were used. Contrary to our findings, results from Jiang et al, who investigated the effect of adipose tissue derived MSCs on neutrophil mediated tissue damage, revealed that mouse derived neutrophils cocultured with MSCs exhibited a decrease in MPO and NE secreted levels as well as their enzymatic activities,^32^ suggesting potential differences between these interactions between human and mouse cells. In another study, human MSCs derived from Wharton's jelly, trabecular bone and BM were capable of suppressing human neutrophil recruitment to TNF‐α‐stimulated endothelial cell monolayers.^33^ As distinct stimulation approaches can be used for neutrophil activation and recruitment, these differences may be useful in evaluating differential responses to stromal cells. In addition, cells derived from several tissue sources such as adipose, perinatal tissues exhibit distinct therapeutic properties and immunomodulatory effects that could impact the interactions with the immune system. Studies have shown that MSCs derived from the umbilical cord exhibit a significantly different paracrine profile when compared to adipose derived MSCs suggesting that the tissue source of MSCs strongly impacts the biological diversity potential of the derived cells and thus influence their immunomodulatory properties.^34^, ^35^ Similarly, the presence of multiple interacting cell types appears to be essential for appropriate modeling of neutrophil functions. Our neutrophil isolation protocol eliminated other immune cell populations such as macrophages and monocytes, suggesting these cell interactions may be important for neutrophil activation and interactions with stromal cells.^36^ It is possible that the observed inhibitory effect on NE gene expression may be mediated by indirect mechanisms, where perinatal cells interact with other components of the immune system which then influence neutrophil functions. This hypothesis requires further study, but is supported by a study performed by Lai et al who attributed the induced NE reduction in a rat model of ventilator‐induced lung injury, to the MSCs suppression of early stage pro‐inflammatory cytokines like TNF‐α.^37^

When we evaluated the inhibitory effects of AFSCs, BM‐MSCs, and PLSCs on PHA stimulated PBMCs from healthy donors, we found that PLSCs suppressed the proliferation of stimulated lymphocytes in vitro as shown in Figure 4. This finding confirms studies performed by others, who have shown that PHA‐stimulated lymphocyte proliferation can be suppressed by bone‐marrow and perinatal‐derived cells under coculture conditions.^23^ Our studies further explored the mechanisms of this phenomenon, using our transwell culture system and cell conditioned media to determine that while PLSCs suppress the gene expression of multiple inflammatory cytokines through secreted factors, direct contact is needed to affect Th1 proliferation. Thus, our study provides novel findings showing that direct cell‐to‐cell contact is required for PLSCs to suppress the proliferation of Th1 cells in our experimental design. Such finding is novel because it elaborates on the potential mechanism of action of PLSCs on Th1 cells and thus provides further understanding on how the use of these cells or their secreted factors may influence specific immune functions. We also demonstrated by flow cytometry analysis of the Th cell subsets that Th1 (CD3^+^ CD4^+^ CD183^+^) proportions (%) were reduced in the presence of PLSCs. This is supported by our observed decrease in levels of secreted IFN‐γ shown in Figure 5. Our findings are in accordance with other reports that showed that PLSCs downregulate Th1 cells and decrease the levels of their associated cytokines such as IFN‐γ and IL‐2.^23^, ^38^, ^39^ Th1 cells mediate immune responses against intracellular bacteria and protozoa by producing IL‐2 and IFN‐γ that lead to activation of macrophages as well as CD8^+^ T cells and antibody producing B cells.^40^ During an infection, antigen presenting cells present antigen derived proteins to naïve T cells (Th0) which recognize these signals using their T cell receptors and become activated. CD4^+^ naïve T cells then differentiate into multiple T helper subsets including Th1. Th0 give rise to Th1 cells in the presence of IL‐12 and IFN‐γ, which trigger the expression of high levels of STAT4 and T‐bet transcription factors in naïve T cells, favoring Th1 differentiation.^41^ Th1 have been implicated in many illnesses as a potential mechanism of disease dysfunction and progression. Studies have shown that increased levels of IFN‐γ, associated with a predominant Th1 imbalanced inflammatory response, play a major role in the progression of autoimmune disease in lupus prone MRL mice.^42^ Other studies investigating the role of Th1 in RA in rat models revealed that polyclonal expansion of Th1 cells was necessary for the onset of disease.^43^ Patients with CD exhibited high infiltration of Th1 cells in the gastric and intestinal mucosa, accompanied by increased levels of IFN‐γ and TNF‐α which have been implicated in the progression of the disease.^44^ In autoimmune demyelinating diseases, such as MS, patients showed elevated serum levels of TNF and IFN‐γ derived from Th1 cells.^45^, ^46^ More recently, Zhou et al reported that patients with psoriasis exhibited increased levels of IFN‐γ as well as IL‐17 in the serum, which were mainly mediated by increased NF‐κB expression.^47^ Current treatments for Th1 mediated diseases include anti‐inflammatory and immunomodulatory drugs to reduce the Th1 mediated immune response, such as anti‐TNFα antibodies.^48^, ^49^, ^50^, ^51^ However, these therapies have been associated with major side effects such as infusion reactions especially in nonspecific cells of the body, and is some cases, the development of eczema and tuberculosis due to neutralization of TNF‐α.^52^, ^53^ Thus, the potential to modulate Th1 responses in patients with autoimmune diseases may be beneficial for controlling or preventing disease onset. Interestingly, the presence of PLSCs in contact with stimulated immune cells led to an increase in IL‐17A and a potential reduction in IL‐2 supernatant levels shown is Figure 6. IL‐2 is mainly involved in the maintenance and growth of regulatory T cells (Tregs), as well as the differentiation of Th1 and Th2 cells. IL‐2 might also play a role in the suppression of the differentiation of Th17 cells, the main sources for IL‐17A. Th17 cells, on the other hand, are highly inflammatory cells that are involved in a number of autoimmune diseases such as psoriasis, RA, and MS.^54^, ^55^, ^56^, ^57^ This may explain our findings showing elevated levels of IL‐17A and reduced IL‐2 levels following PHA stimulation. However, the administration of PLSCs further exacerbated this phenomenon, suggesting that PLSCs may favor Th17 cells. In this context, although we did not detect any significant changes in the percentage of Th17 cells in our coculture system with PLSCs, it seems that the presence of PLSCs might increase the function of these cells to produce IL‐17A. This suggests that the suppressive effect of PLSCs on Th1 cells might create a suitable environment for Th17 cells upregulation. This contradicts some of the reports showing that PLSCs may suppress the expansion of Th17 cells.^23^, ^39^ Along the lines of our results, MSCs activation of Th17 has been previously reported in vitro, as Carrion et al demonstrated similar opposing effects on Th1 and Th17 cells, where the addition of MSCs after T cell activation led to an increase in IL‐17A production while inhibiting IFN‐γ. MSCs only decreased Th17 cells when added early (prior to T cell activation).^58^ These findings emphasize the importance of investigating the underlying mechanisms of action of cell therapy and its effect on the different cells of the immune response, taking into consideration important criteria such as the state of T cell activation as well as timing of cell therapy administration. PLSCs' suppression of one axis of the pro‐inflammatory response might come at the risk of allowing upregulation of other inflammatory pathways that could be just as deleterious to disease severity.

## 5 CONCLUSION

Our study showed that PLSCs have a robust effect on the suppression and function of Th1 subsets. These findings confirm the anti‐inflammatory properties of PLSCs on Th1, provide further understanding approximately the interaction of PLSCs with the different components of the immune system, and offer a potential anti‐inflammatory effect on Th1 cell subset, suggesting a potential use of these cells to target Th1‐related diseases.

## CONFLICT OF INTEREST

The authors indicated no potential conflicts of interest.

## AUTHOR CONTRIBUTIONS

O.K.: conception and design, collection and/or assembly of data, data analysis and interpretation, manuscript writing, final approval of the manuscript; A.A., S.V.M.: financial support, conception and design, data analysis and interpretation, manuscript writing, final approval of the manuscript.

## Supporting information


**Supplemental Figure 1** Secreted levels of IL‐6 from unstimulated (US) and LPS exposed AFSCs, BM‐MSCs and PLSCs. Bars represent SEM.

## Data Availability

The data that support the findings of this study are available from the corresponding author upon reasonable request.
