# Supplementary figures and images for: Stromal cells from perinatal and adult sources modulate the inflammatory immune response in vitro by decreasing Th1 cell proliferation and cytokine secretion
Source: Stem Cells Transl Med. 2019 Oct 22;9(1):61–73. doi: 10.1002/sctm.19-0123 (PMC6954711; doi:10.1002/sctm.19-0123)

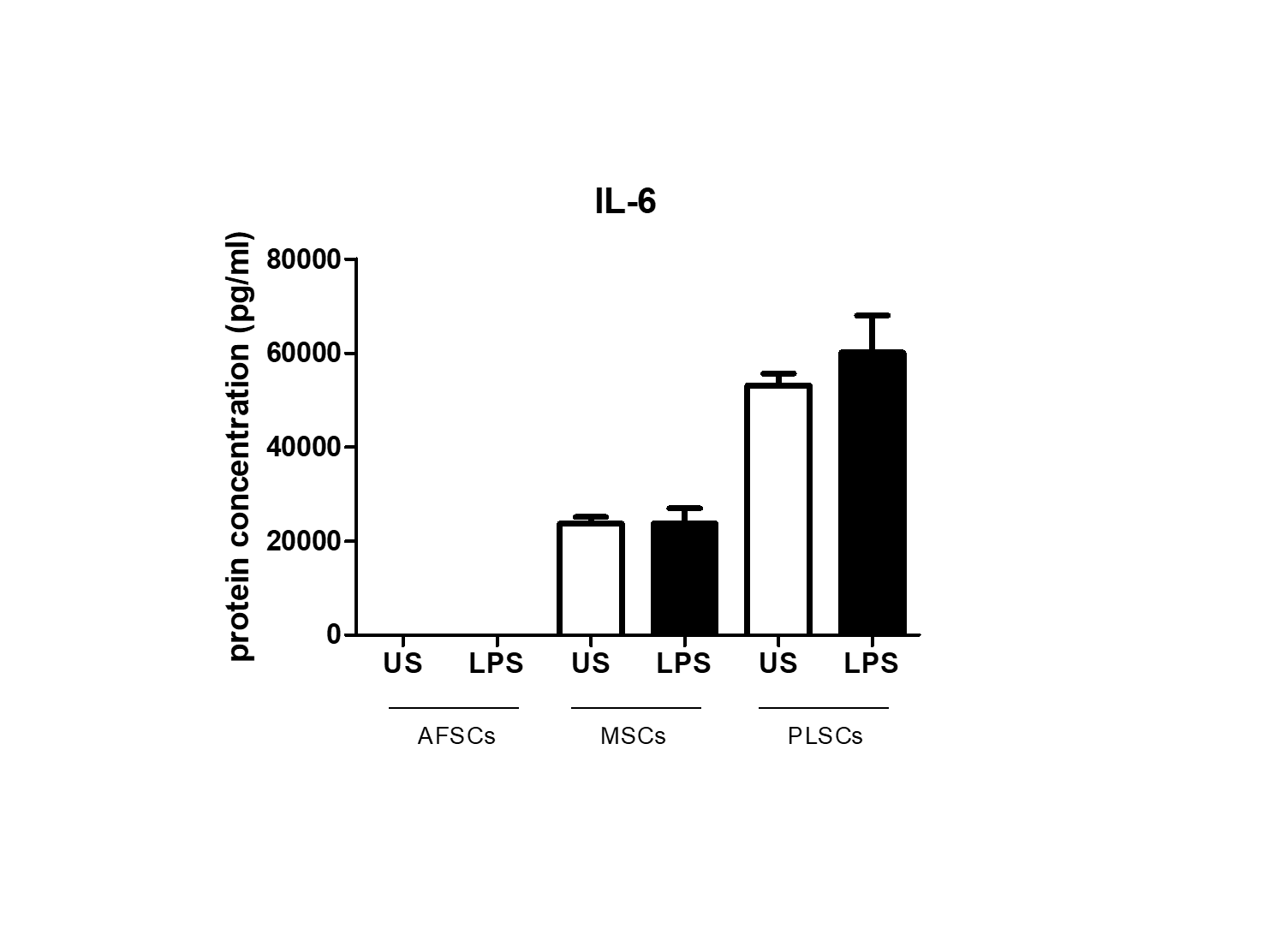

Supplement: Supplementary file 1 — Supplemental Figure 1 Secreted levels of IL‐6 from unstimulated (US) and LPS exposed AFSCs, BM‐MSCs and PLSCs. Bars represent SEM. [file SCT3-9-61-s001.tif]
